# MusicAlzheimer: AI-Powered, Culturally Tailored Digital Music Therapy Prototype for Alzheimer Disease Care

**DOI:** 10.2196/89535

**Published:** 2026-05-13

**Authors:** ShuRan Yang, Daniel YunShu Zhang, MinMin Zhang, Hua Yang, YongHui Ma

**Affiliations:** 1 Shanghai Foreign Language School Affiliated to Shanghai International Studies University, Shanghai, China; 2 Shanghai High School International Diversion, Shanghai, China; 3 Department of Gastroenterology/Endoscopy Center, Ruijin Hospital, Shanghai JiaoTong University, School of Medicine, Shanghai, China; 4 Department of Geriatrics Neurology, Haowangjiao Hospital, Shanghai, China; 5 Teaching Department, Franklin Academy, 27442 Portola Pkwy, Ste 150, Lake Forest, CA, 92610, United States

**Keywords:** Alzheimer disease, digital music therapy, artificial intelligence, culturally tailored intervention, caregiver support, nonpharmacological intervention, dementia care

## Abstract

This research letter reports the development and preliminary user testing of *MusicAlzheimer*, an artificial intelligence–driven digital music therapy prototype designed to deliver culturally tailored, real-time adaptive interventions for people with Alzheimer disease.

## Introduction

Alzheimer disease (AD) is a leading cause of dementia [1]. Symptoms of dementia, including agitation, psychosis, depression, and apathy, impact patients’ quality of life, disrupt relationships, increase caregiver burden, and elevate patients’ risks of institutionalization and mortality [2]. Nonpharmacological interventions such as music therapy (MT) have potential to reduce agitation, support emotional stabilization, and leverage auditory-cognitive pathways to stimulate autobiographical memory and modulate emotional responses [3-5]. The therapeutic effect of MT is enhanced when culturally familiar music is used [6]. However, traditional MT interventions face significant limitations and lack dynamic personalization and real-time adaptation to patients’ emotional and cognitive states, limiting their overall effectiveness.

To solve these critical problems, we developed *MusicAlzheimer*, an artificial intelligence–powered digital MT platform. It integrates culturally tailored music-based interventions with adaptation to real-time emotional states of patients and personalization measures.

## Methods

### System Design

**Figure 1.**
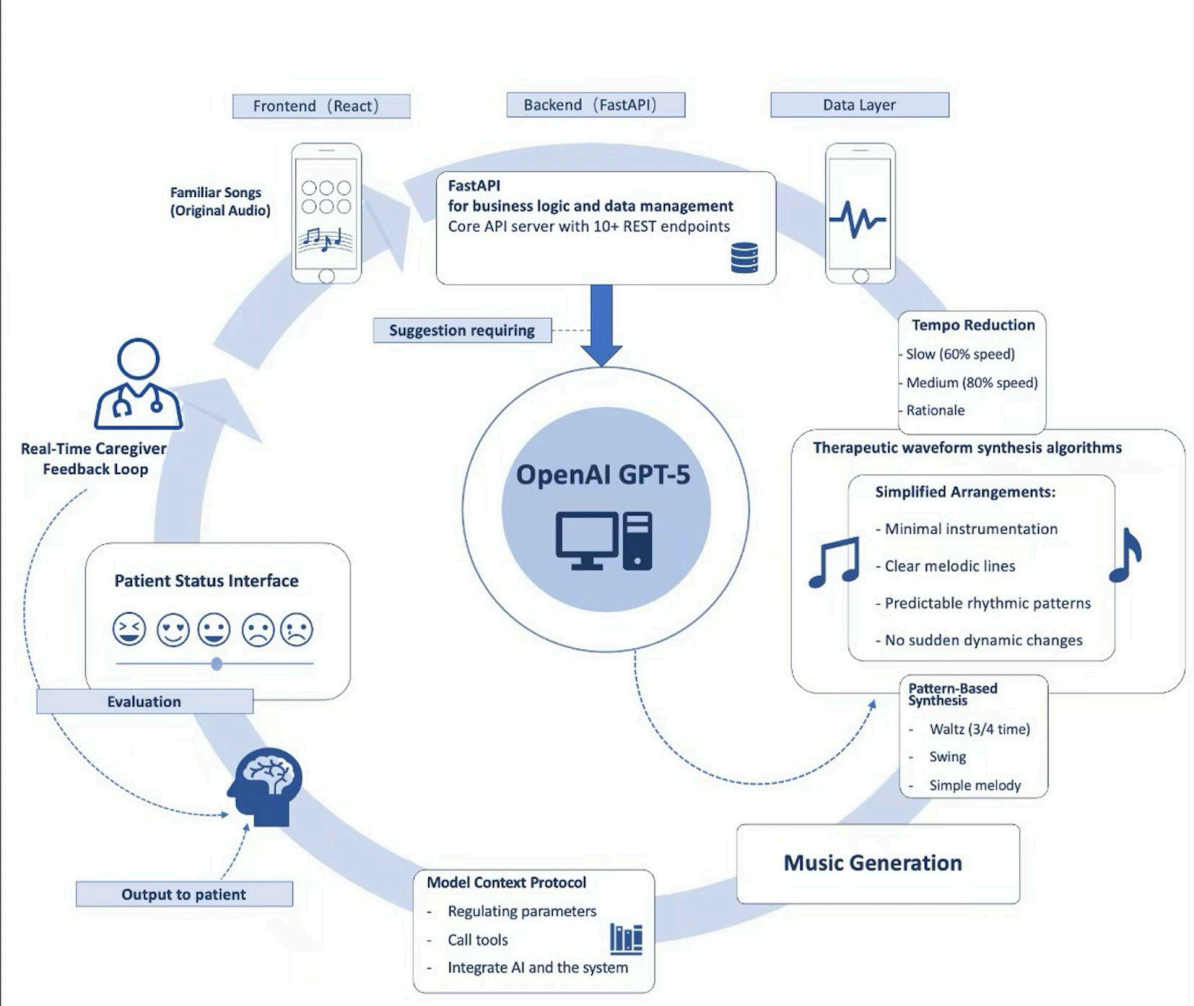
hereProject architecture of the *MusicAlzheimer* system.

The *MusicAlzheimer* prototype includes (1) a culturally adapted therapeutic music library (18 tracks across 3 categories), (2) a GPT-supported caregiver prompt engine, and (3) an emotional-tracking and analytics interface (Figure 1).

During use, caregivers manually recorded patients’ emotional states (distressed, neutral, content, joyful) and memory-response cues (None, Some, Strong) through a web-based interface. These inputs informed adaptive music selection and generation of concise (<20 word), context-aware caregiver prompts. All session-level interactions were stored locally to ensure privacy (on-device SQLite database).

### Participants and Usability Evaluation Procedure

A convenience sample of 8 informal caregivers of patients with mild-to-moderate AD participated in structured usability sessions. Participants were recruited through local community networks and caregiver support groups. Each usability session was conducted individually on a laptop provided by the researchers and comprised three phases:

- System orientation (~10 min): A brief introduction to the interface, covering music playback controls, mood state input, and the GPT-5 recommendation panel.
- Simulated or live therapy session (~20 min): Caregivers operated the *MusicAlzheimer* system with their care receivers present (n=5) or with a researcher simulating the patient role (n=3).
- Structured postsession feedback (~10 min): Participants completed a paper-based questionnaire with default response categories across 4 domains (music quality perceived and patient accessibility; patient behavioral responses observed; utility of GPT prompts; overall system acceptability).

No validated scales (eg, system usability scale) were used during this formative evaluation phase. No patient health-related data were collected or stored at any stage during the study.

### Ethical Considerations

This study involved only adult informal caregivers providing usability feedback on a nonclinical prototype system. The study was classified as exempt from nonclinical software usability assessment in accordance with local institutional policy. All participants provided verbal informed consent, including for data use and storage. Formal institutional review board approval was not required.

### System Modules

#### Module 1: System Initialization and Music Library

Core system components were loaded during initialization. Audio files (WAV format), metadata (tempo, key, category), and session flow schemas were parsed by a FastAPI module. The React front end loaded user interface state schemas for emotional-state and memory-response tracking (Multimedia Appendix 1).

Tracks were organized across 3 evidence-based therapeutic categories: Familiar (1940s-1970s songs designed to trigger autobiographical memory recognition), Soothing (slow tempo, gentle harmonics for agitation reduction), and Uplifting (moderate tempo, major-key tonality for mood elevation). Simplified therapeutic versions featured reduced tempos (60%‐80% of original speed) and pattern-based synthesis styles (waltz, swing, simple melodic lines), optimized for cognitive accessibility for AD.

#### Module 2: Embedding Generation

To support real-time music adaptation and context-aware caregiver guidance, all therapeutic tracks and conversational prompt templates generate embedded context and conversational prompt templates. Audio embeddings captured timbre, tempo, and emotional characteristics. Text embeddings for caregiver prompts and cultural-era descriptors were generated through the MCP server’s integrated local pipeline, which makes the semantic retrieval of contextually appropriate tracks and prompts at runtime possible.

#### Module 3: Real-Time Session Processing

During sessions, the system executed a multimodal retrieval and inference workflow. Through WebSocket channels, the React front end transmitted real-time emotional state updates and memorial response levels to the FastAPI back end. The MCP server coordinated GPT-5 to select the caregiver’s prompts and memory cues. GPT-5 produced context-aware guidance (<20 words) tailored to the patient’s current emotional state, selected music, and previous session history (Table 1).

**Table 1.** Examples of caregiver prompts across all four emotional state categories generated by GPT-5.

Emotional state	Track category	Example GPT-5 caregiver prompt (<20 words)	Behavioral tip
Distressed	Soothing	Do you remember hearing this waltz at a summer dance?	Maintain gentle eye contact; speak slowly and softly.
Neutral	Familiar	This melody was popular when you were young, wasn’t it?	Invite the patient to tap along gently with the rhythm.
Content	Uplifting	Does this remind you of a favorite film from the 1950s?	Encourage verbal reminiscence; listen without interrupting.
Joyful	Familiar	I remember you loved music like this — tell me more.	Allow the patient to lead the conversation naturally.

#### Module 4: Session Logging and Analytics

All interactions were written to an on-device SQLite database with time stamps, track metadata, behavioral observations, and caregiver notes. The analytics module visualized longitudinal patterns, identified high-response tracks, and summarized weekly engagement trends.

## Results

Caregiver testing demonstrated that *MusicAlzheimer*’s modules functioned cohesively to support personalized dementia care. Findings are organized into 4 domains.

### Music Quality and Patient Accessibility

All 8 caregivers described the automatically generated music as familiar and easier for patients to process. Seven rated the music as calming. Simplified therapeutic tracks with reduced tempos were perceived as more accessible and more likely to evoke emotional resonance.

### Observed Patient Behavioral Responses

Caregivers reported increased patient engagement when culturally relevant tracks were used. Seven observed calming effects. Six caregivers reported improved eye contact or sustained attention and reduced agitation in patients. Five patients exhibited verbal reminiscence. The system’s dynamic music adaptation addressed long-standing limitations of traditional MT, such as static playlists and lack of personalization.

### GPT-5 Caregiver Prompt Module

All caregivers rated the GPT-based caregiver module as very useful. It provided short, context-sensitive cues that caregivers found easy to incorporate into ongoing interactions. These prompts were described as helpful for maintaining engagement during moments of distraction or emotional fluctuation.

### Session Analytics Interface

The interface allowed caregivers to review emotional trends and identify music patterns associated with stronger engagement. Seven caregivers reported that the longitudinal trend visualization helped them better understand individual patient responses and informed music selection in subsequent sessions.

### Overall System Acceptance

Seven caregivers reported being able to operate the system independently after the orientation phase. They reported minimal learning burden and that they can better understand individual patient responses. Collectively, early feedback highlights the feasibility and potential utility of such a system.

## Discussion

GPT-generated prompts in *MusicAlzheimer* were intentionally concise and era-specific to support reminiscence and help caregivers sustain engagement during sessions, helping reduce caregivers’ burden and support more consistent therapeutic engagement.

The platform leverages preserved auditory and affective pathways in AD by pairing culturally familiar music with personally relevant cues, which may facilitate emotional regulation and memory recall [7-9]. Such multimodal stimulation can reduce the anxiety or confusion that often interfere with recall, enhancing the therapeutic impact of familiar music interventions. The robust emotional-tracking component captures basic affective states and memory-response levels, offering caregivers simple trend insights during home-based sessions.

Regarding potential longer-term cognitive effects, two theoretical frameworks provide relevant support. First, MT can influence neural plasticity. By stimulating the remaining auditory and emotional neural networks continuously, MT can strengthen the integrity of neural pathways and help regulate the emotions of patients with AD when the disease progresses [10]. Second, music can trigger both direct (music directly evokes specific memories) and indirect retrieval (music can create a mood to facilitate the retrieval of a broader range of memories). *MusicAlzheimer* combines era-specific music with GPT-generated language to engage both pathways simultaneously [11,12]. We recommend using validated outcome measures, such as the Mini-Mental State Examination and the Alzheimer’s Disease Assessment Scale-Cognitive Subscale, to form longitudinal evaluations before making any claims regarding efficacy.

Study limitations include a small sample size, limited culturally diverse music, reliance on subjective caregiver ratings rather than validated clinical instruments, simplified audio synthesis, dependence on internet connectivity, and the absence of clinical outcome validation. As a prototype-stage feasibility study, the system is not intended to replace professional therapy but to complement dementia care for individuals with preserved musical memory. Future work will expand multimodal sensing, diversify musical content, evaluate clinical impact in controlled studies, and include on-device large language model deployment for offline capability.

## Supplementary material

10.2196/89535 Multimedia Appendix 1 Examples of simplified therapeutic audio outputs and the caregiver interaction interface. (A) Interface allowing the user to select requirements for music generation from the MusicAlzheimer system; (B) the music library of the *MusicAlzheimer* system; (C) interface of the session tracking of the *MusicAlzheimer* system.

## Abbreviations

AD: Alzheimer disease
MT: music therapy
