# Supplementary figures and images for: MusicAlzheimer: AI-Powered, Culturally Tailored Digital Music Therapy Prototype for Alzheimer Disease Care
Source: JMIR Form Res. 2026 May 13;10:e89535. doi: 10.2196/89535 (PMC13170998; doi:10.2196/89535)

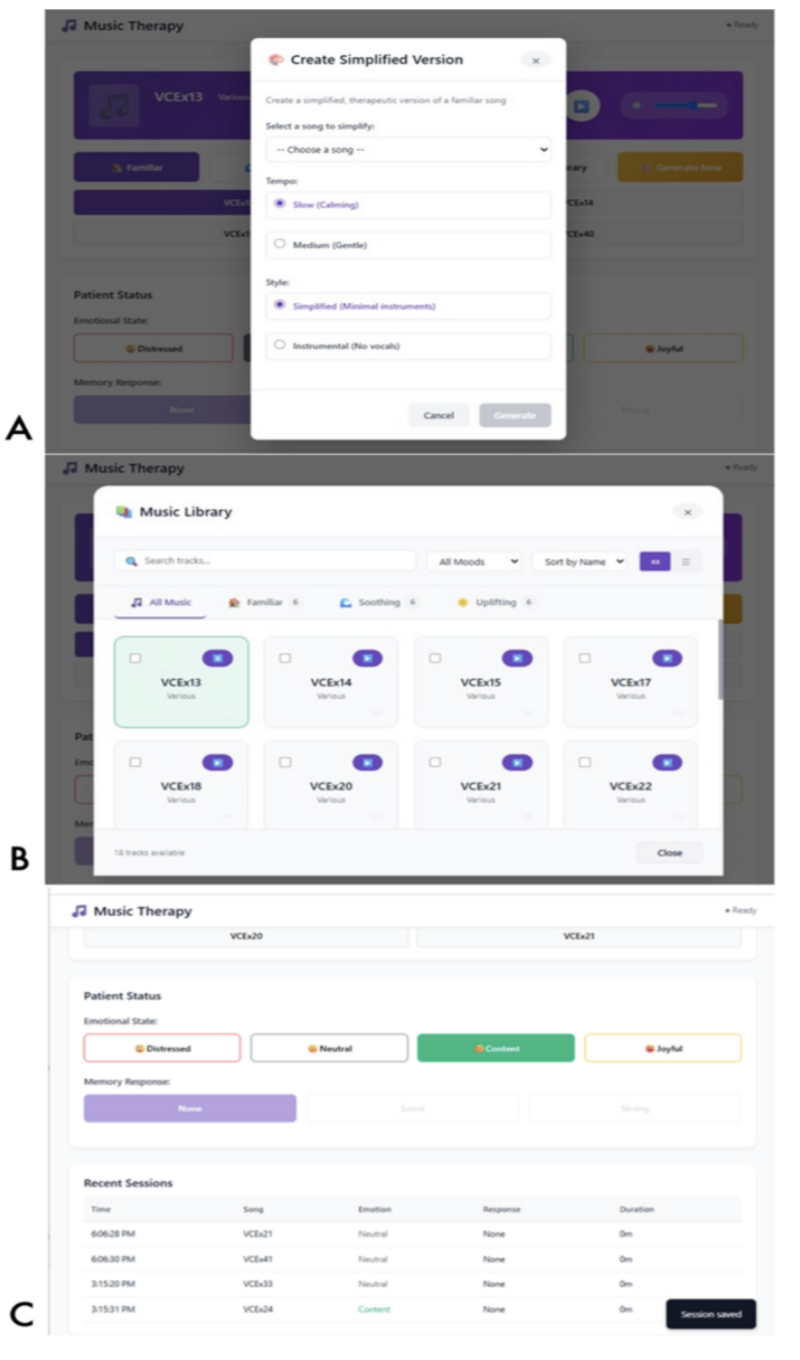

Supplement: Multimedia Appendix 1 [file formative-v10-e89535-s001.png]
